# Preliminary comparative analysis of the genomes of selected field reisolates of the *Mycoplasma synoviae* vaccine strain MS-H reveals both stable and unstable mutations after passage in vivo

**DOI:** 10.1186/s12864-020-06995-z

**Published:** 2020-08-28

**Authors:** Somayeh Kordafshari, Pollob Shil, Marc S. Marenda, Olusola M. Olaogun, Barbara Konsak-Ilievski, Jillian Disint, Amir H. Noormohammadi

**Affiliations:** Asia Pacific Centre for Animal Health, Faculty of Veterinary & Agricultural Sciences, The University of Melbourne, Werribee, Victoria 3030 Australia

**Keywords:** *Mycoplasma synoviae*, MS-H vaccine strain, Genomic comparison, Stable and unstable mutations, MS-H field reisolates

## Abstract

**Background:**

Genomic comparison of *Mycoplasma synoviae* vaccine strain MS-H and the MS-H parental strain 86,079/7NS established a preliminary profile of genes related to attenuation of MS-H. In this study we aimed to identify the stability of mutations found in MS-H after passage in experimental or field chickens, and to evaluate if any reverse mutation may be associated with changes in characteristics of MS-H in vitro or in vivo.

**Results:**

Whole genome sequence analysis of 5 selected MS-H field reisolates revealed that out of 32 mutations reported previously in MS-H, 28 remained stable, while four found to be reversible to the wild-type. Each isolate possessed mutations in one to three of the genes *obg*, *oppF*_*1*_ and *gap* and/or a non-coding region. Examination of the 4 reversible mutations by protein modeling predicted that only two of them (in *obg* and *oppF*_1_ genes) could potentially restore the function of the respective protein to that of the wild-type.

**Conclusions:**

These results suggest that the majority of the MS-H mutations are stable after passage in vaccinated chickens. Characterisation of stable mutations found in MS-H could be utilised to develop rapid diagnostic techniques for differentiation of vaccine from field strains or *ts-* MS-H reisolates.

## Background

*Mycoplasma synoviae* (MS) is a major poultry pathogen, and due to its high economic impact on sectors of the chicken and turkey industries [1] has been listed as a serious disease of poultry by the World Organization for Animal Health (OIE, http://www.oie.int/animal-health-in-the-world/oie-listed-diseases-2019/). Control of the disease caused by MS through biosecurity and serological monitoring is often insufficient [2]. Therefore, live attenuated vaccines are used when the prevention of exposure is impractical. The most commonly used commercial MS vaccine in Australia (Vaxsafe MS®; Bioproperties Ltd., Ringwood, Victoria, Australia) is a temperature sensitive (*ts+*) strain (MS-H) which was developed by chemical mutagenesis of an Australian field isolate 86,079/7NS [3].

A wide range of *ts +* viruses and bacteria have been used as vaccine candidates, but in many cases it is not exactly known whether temperature sensitivity is the cause of attenuation or just a coincidental phenotype in these organisms [4, 5].

The majority of MS clones recovered from vaccinated flocks display their *ts +* phenotype, but it has been suggested that MS-H proliferation in vaccinated birds generates a mixture of *ts +* and *ts-* clones in the farm [6, 7].

Unlike the non-virulent MSH strain, *ts-* field reisolates cause only minor lesions in the tracheal mucosa of the experimentally infected birds, significantly lower than the vaccine parent strain [7]. These results suggest that factors other than *ts +* phenotype are involved in the attenuation of the MS-H vaccine.

While the genetic basis of the MS-H temperature sensitivity and attenuation is not fully known yet, a mutation detected in *obg* gene was proposed as a likely explanation for the MS-H *ts +* phenotype [8]. Also, further comparison of the MS-H genome with that of its wild-type parent strain 86,079/7NS has revealed a frameshift mutation in an oligopeptide permease transporter (*opp*) gene, *oppF*_*1*_ [9]. OppF is essential in establishment of systemic infection by *M. bovis* and its persistence in lower respiratory tract of calves [10]. Also, *oppD* was found to be required for full expression of virulence of *M. gallisepticum* in chickens [11].

Partial sequence analysis of *obg* and *oppF* genes [8, 12] in five MS-H isolates have found different combinations of *obg* and *oppF* genotypes. Of the five isolates, MS-H^3^, 101,564 and 101,731 had *obg*^w^ (w = wild-type) and *oppF*^v^ (v = vaccine-type), MS-H^4^ had *obg*^w^ and *oppF*^w^, and MS-H^5^ had *obg*^v^ and *oppF*^w^. In this study the MS-H reisolates MS-H^3^, 101,564, 101,731 MS-H^4^, and MS-H^5^ were subjected to a comparative genome analysis to establish if any other mutations previously reported for the MS-H [12] may be reversible to the wild-type and evaluate if they could potentially influence MS-H attenuation.

## Results

### Phylogenetically, all selected reisolates from vaccinated flocks were closely related to MS-H

Illumina paired reads from MS-H field isolates (GenBank accession number PRJNA649354) were De novo assembled successfully using SPAdes with an average 162 of contigs generated for each ranging from 125 to 131,331 bp per isolate. The *vlhA* pseudogene region, a ~ 50 kb locus covering large number of highly repetitive sequences, as well as the repetitive and the highly similar IS failed to assemble. Otherwise, the SPAdes generated an average 790,468 bp, representing a complete genome with high identity (93%) to other MS sequences available in the Gene Bank [12–16]. Alignment of the draft genomes of MS-H field isolates with that of MS-H exhibited an overall high degree of sequence similarity (99.99%) with no large-scale chromosomal insertions, deletions, duplications or rearrangements except for *vlhA* locus.

**Fig. 1:**
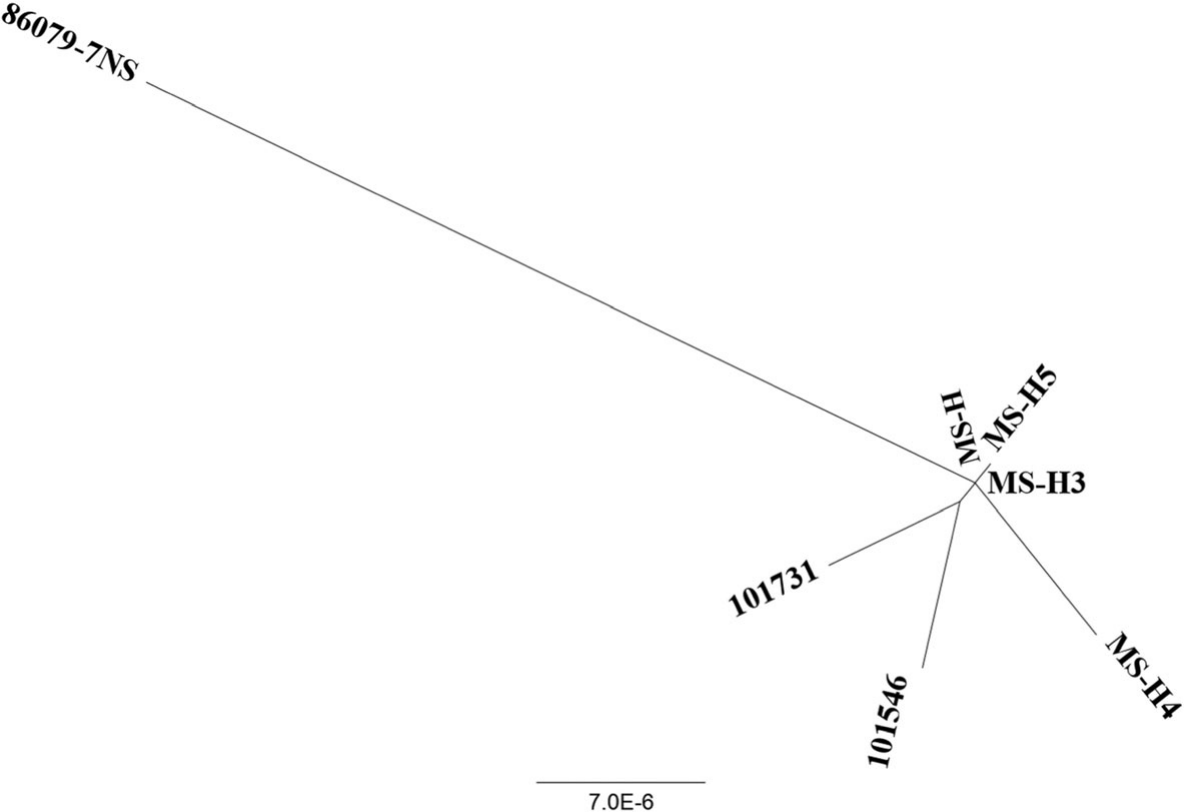
Phylogenetic tree from seven MS strains/isolates. The phylogenetic tree was inferred from whole genome comparison of seven MS strains/isolates using Neighbor Joining (NJ) and Maximum likelihood methods. The scale bar shows the distance

The maximum likelihood and NJ analysis performed using platforms REALPHY and MEGA, respectively, on whole genome sequences of 7 MS strains/isolates generated highly comparable results, reflecting a close relationship between MS-H and its field isolates. Notably, MS-H^3^ and MS-H^4^ were respectively the most closely and distantly related to MS-H (Fig. 1).

### Of the 32 mutations previously found between 86,079/7NS and MS-H, only four were observed to have reversed

Comparative genomic analysis found a total of 25 SNP and indel variants between MS-H and its field isolates (Table 1). MS-H^4^ and MS-H^3^ had the highest (12) and the lowest (1) number of genomic differences with MS-H, respectively, while MS-H^5^, 101,546 and 101,731 had 4, 7 and 7 differences, respectively. Four out of these 25 SNPs had been detected in a previous study that compared the genomes of MS-H and its parent strain 86,079/7NS [12], however the other 21 were found only in the 5 reisolates.

**Table 1:**
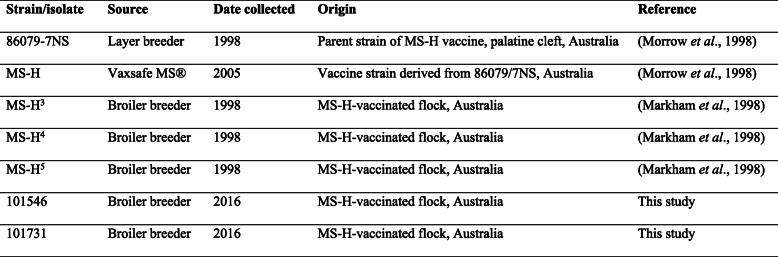
Nucleotide and coding differences identified among the genomes of 86,079/7NS, MS-H and 5 MS-H field isolates

The MS-H^3^ had an identical sequence to MS-H except for the coding DNA sequence (CDS) of *oppF* in which insertion of ‘T’ at position 468 resulted in the restoration of the full-length *oppF* sequence identical to that of 86,079/7NS.

Three non-synonymous differences were found between genomes of MS-H and MS-H^5^. These included deletion of two nucleotides ‘AT’ in a tandem repeat in a non-coding region (positions 502,825) upstream of the cytosine-5-methyltransferase CDS, insertion of nucleotide ‘A’ (causing frame-shift mutation) in a gene (CDS 164) encoding a protein of unknown function, and nucleotide substitution ‘A’ to ‘G’ in *obg* gene resulting in restoration of the wild-type Obg (Arg123Gly). Also, a synonymous substitution (‘C’ to ‘T’) in Glu322 was found in the CDS 966 which codes for Desert Hedgehog Signalling Molecule.

Twelve genomic differences were found between MS-H^4^ and MS-H, three of which had been described to exist between MS-H and 86,079/7NS and were reverted to wild-type sequence. These comprised of insertion of ‘AT’ at position 502,827 in a tandem repeat within a non-coding region, a SNP in *obg* gene (similar to that found in MS-H^5^) and a frameshift mutation in the *oppF* gene (identical to that found in MS-H^3^). The other 9 mutations comprised of 6 in genes coding for Cardiolipin, two hypothetical protein, TatD deoxyribonuclease, S1 RNA-binding domain, and Thymidine phosphorylase, 1 in a gene with unknown function, 2 in non-coding regions.

In the isolate 101,546, two genomic differences were found to cause reversion to wild-type sequence. These included ‘A’ to ‘G’ in *gap* gene (at CDS 554) which resulted in a conservative change (Ala185Val), and a frameshift mutation in *oppF* identical to that from MS-H^3^ and MS-H^4^. Moreover, four non-synonymous substitutions were found in CDSs corresponding to Obg, YbhB/YbcL Raf kinase inhibitor, and two hypothetical proteins. These substitutions were due to ‘C’ to ‘T’ at CDS 629 in *obg* gene resulting in a conservative change (Ala210Val); ‘C’ to ‘A’ at CDS 909 causing a conservative change (Asp303Glu) in a gene encoding a hypothetical protein; ‘G’ to ‘A’ at CDS 220 resulting a conservative change (Val74Ile) in gene encoding YbhB/YbcL Raf kinase inhibitor, and ‘C’ to ‘T’ at CDS 3979 resulting in a non-conservative substitution (Ala1327Thr). Moreover, a synonymous substitution in Thr197 was found in CDS corresponding to LemA (‘C’ to ‘T’ at CDS 591).

Comparison of MS-H and 101,731 were found changes in *obg* and *oppF* genes consistent with those of 1,015,465. Moreover, similar to MS-H^4^, ‘AT’ insertions at positions 502,827 was found resulting in reversion to wild-type sequence. In addition, a ‘T’ deletion at CDS 387 in a gene encoding a hypothetical protein, substituted Tryr135 to a premature stop-codon. Moreover, genes encode Cls and DNA-directed RNA polymerase subunit beta were found to have a ‘G’ to ‘A’ substitution which resulted in a conservative amino acid change (Ser263Asn), and a ‘T’ to ‘C’ substitution which resulted in a non-conservative substitution (Glu1037Gly), respectively. Additionally, a synonymous substitution in Asx362 was found in CDS corresponding to a hypothetical protein (‘G’ to ‘A’ at CDS 1086).

### Mutations found in genes coding for Obg, OppF, Cardiolipin, and YbhB/YbcL Raf kinase-inhibitor were computationally predicted to affect the proteins structure

The final alignment between targeted proteins and templates using Phyre2 for eight proteins (OppF, Obg, Cls, TatD, S1 RNA-binding, NAD-dependent glyceraldehyde-3-phosphate dehydrogenase, YbhB/YbcL Raf kinase inhibitor and DNA-directed RNA polymerase subunit beta) exhibited an average 76% of residues modelled at 100% confidence. For the other five proteins (hypothetical proteins) only an average 40% of residues modelled at > 50% confidence and none of residues modelled at > 90% confidence (Table 2). The model template IDs, protein lengths, predicted secondary structures, and the degree (%) of mutation sensitivity at a given position in the respective proteins are detailed in Table 2.

**Table 2:**
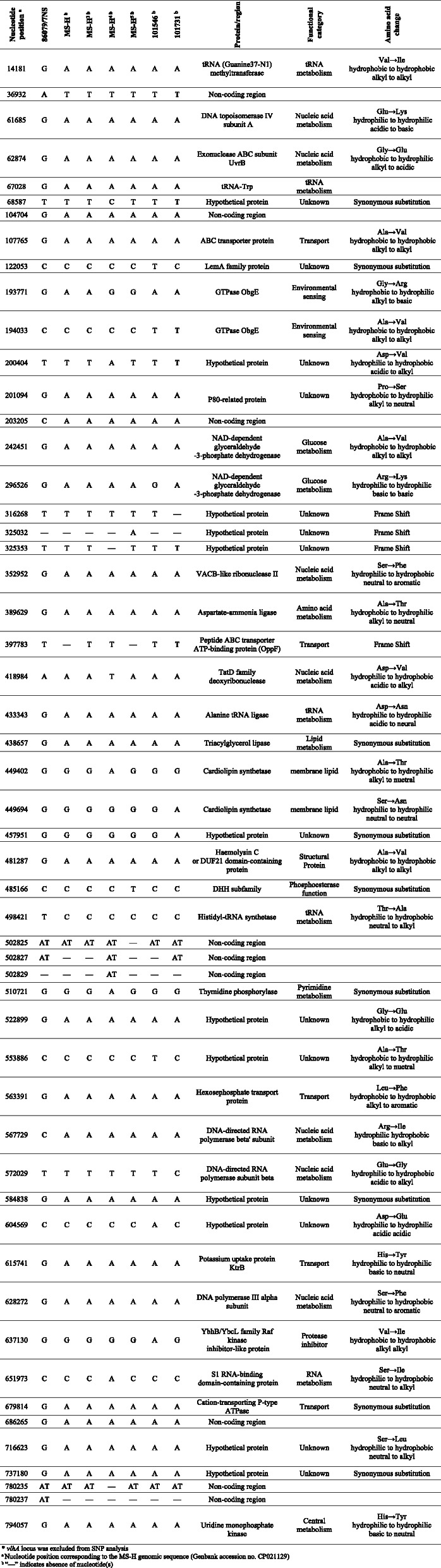
Homology modelling results of proteins vary between MS-H and its field isolates

Consistent with findings from a previous study [8], homology modelling of Obg in MS-H showed that Arg123 is located in large pocket region, which are frequently the active sites [17], however the probability that a missense mutation at this position effecting function of the corresponding protein was predicted low. The percentages of predicted alpha helices in the Obg protein sequences of MS-H^4^ or MS-H^5^ (29%), was different from that of MS-H (26%), due to the Arg123Gly difference between these strains. By contrast, the Ala210Val difference in the Obg protein sequence of strains 101,546 and 101,731 did not change the secondary structure of Obg compared to that of MS-H.

The frameshift mutation corresponding to *oppF* gene in MS-H^3^, MS-H^4^, 101,546 and 101,731 restored the full-length OppF (reversion to wild-type). Based on the protein homology analysis conducted as part of this study, the functional domain of OppF is identified at the C terminus. As a result, the secondary structure of OppF in above-mentioned isolates (which possessed 68% alpha helices and 7% beta strands) was significantly different to that of MS-H (31% alpha helices and 24% beta strands).

Residue 166 in Cls of MS-H is in a highly sensitive mutation region and therefore Ala166Thr in MS-H^4^ was predicted to affect its function. Also, due to this amino acid change, the secondary structure of Cls in MS-H^4^ (containing 51% alpha helices) was different to that of MS-H (containing 50% alpha helices). By contrast, amino acid at position 263 was in a low mutation sensitive region. Therefore, Ser263Asn was unlikely to affect the protein Cls function in isolate 101,731. Also, the secondary structure of Cls in 101,731 was modelled identical to that from MS-H.

The TatD deoxyribonuclease and S1 RNA-binding proteins were also modelled and compared between MS-H and MS-H^4^ for their mutations at positions 143 and 57, respectively. In MS-H both these mutations were in low sensitive mutation regions and therefore were unlikely to affect the function of the respective proteins in MS-H^4^. However, the latter substitution resulted in a slight change in the percentage of alpha helices from 55% in MS-H to 56% in MS-H^4^, and this could potentially alter the secondary structure of the protein.

The Arg185 in NAD-dependent glyceraldehyde-3-phosphate dehydrogenase in MS-H was in a low mutation sensitive region. The Arg185lys could potentially change the secondary structure of the protein in 101,546 compared to that of MS-H as the percentage of beta strands changed from 31% in MSH to 32% in 101,546.

The protein YbhB/YbcL Raf kinase inhibitor was also modelled in MS-H and 101,546. Residue Val74 was found in a highly sensitive mutation region and therefore Val74Ile could potentially affect the function of the respective protein in 101,546. The secondary structure of this protein was identical in MS-H and 101,546.

The effect of Glu1037Gly in DNA-directed RNA polymerase subunit beta in 101,731 was found neutral as Glu1037 was located in a low sensitive mutation site and the secondary structure of respective protein was identical in MS-H and 101,731.

### Full-length OppF was detected in all MS-H reisolates

**Fig. 2:**
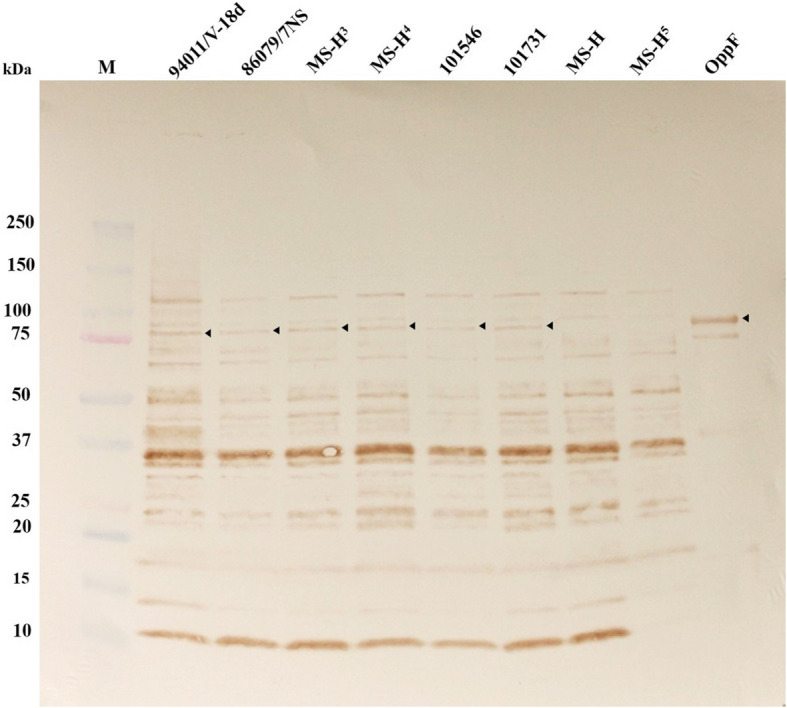
Analysis of OppF expression in MS strains/isolates**.** Western Immunoblots of recombinant purified OppF and whole-cell lysate from MS strains/isolates probed with rabbit-anti-OppF-N. The arrow heads show the location of full-length OppF. M is Precision Plus protein TM, Dual Color marker (Bio-Rad)

Amongst all mutations detected in MS-H reisolates, the frameshift mutation in the *oppF* gene appeared to have the most significant impact on the structure of its encoded protein and therefore was further investigated. The wild-type *oppF* was predicted to encode a polypeptide of 797 amino acids (approximately 94 kDa). Immunoblotting experiments with rabbit-anti-OppF-N antibodies detected the OppF protein of expected size (~ 94 kDa) in 86,079/7NS, MS-H^3^, MS-H^4^, 101,546 and 10,173,118, while did not detect any protein of similar size in MS-H and MS-H^5^ cells (Fig. 2). The rabbit-anti-OppF-N antibodies also detected several presumably nonspecific bands of similar sizes in all MS strains/isolates lysates tested.

## Discussion

This is the first study that investigates the stability of all mutations in a live attenuated mycoplasma vaccine after in vivo passage under field conditions. The initial swab cultures collected from MS-H vaccinated birds were passaged three times in vitro by selection of an individual colony from each step. It may be possible that in vitro passage of the clones may have incorporated selection pressure and bias into the expansion of a clonal population, however it is notable that the clones were compared against an in vitro propagated MS-H vaccine strain. Whole genome sequencing directly from clinical materials collected from vaccinated birds would be ideal to circumvent the potential of in vitro selection pressure, but currently available does not allow compilation of complete genome sequence reliable at a base pair level. Also, current techniques may run the risk of generating a chimeric genome generated from multiple clonal populations that may cohabit the bird’s respiratory system.

A recent study [12] described 32 mutations within the MS-H genome as compared to its parent strain 86,079/7NS. However, the stability of these mutations after passage in vivo had only been tested for those found in *obg* [8] and *oppF* [9].

Protein homology modelling found that four sequence variations between MS-H and reisolates from vaccinated flocks, located in genes coding for Obg, OppF, Cardiolipin, and YbhB/YbcL Raf kinase-inhibitor, were likely to affect the in vitro and/or in vivo characteristics of MS.

Given that *oppF* is involved in pathogenesis of *M. bovis* [10], and the wild-type OppF from MS shares 43% amino acid similarity with that of *M. bovis*, the genomes of five MS-H field isolates differing in the *oppF* gene with that from MS-H and 86,079/7NS was analysed in this study to reflect the possible role of *oppF* in temperature sensitivity/attenuation phenotype of MS-H. Mycoplasmas can survive in vivo due to complex interaction between the microorganism and the host environment [18]. A continuous source of a nutrient used by a gene that is essential for in vivo survival may be a vital factor in the capability of a pathogen to cause disease [10]. Several nutrients are gained from exogenous sources by mycoplasmas as a result of their limited synthesis pathways. Hence, the ability to integrate molecules over membrane-associated transport systems appears to be a substantial factor for in vivo survival of mycoplasmas. In *M. bovis*, two transporters (oligopeptide transporter *oppABCDF* and an uncharacterized transporter) were essential for colonization on the tracheal mucosa [10]. In *M. mycoides* subsp. *mycoides*, a glycerol transporter (*gtsABC*) has been specified as a virulence factor related with hydrogen peroxide production and induction of cytotoxicity [19–21]. The level of mRNA expression of *oppD* of *M. hyopneumoniae* was moderately up-regulated throughout in vivo infection [22] and under iron-depletion conditions [23]. Therefore, all available studies on the role of OppF in several *Mycoplasma species* are highly suggestive that OppF has a major contribution to the attenuation of MS-H. It is notable that in Western Immunoblot analysis conducted as part of this study, the truncated OppF was not delectable in MS-H and MS-H^5^ (Fig. 2). It is speculated that the truncated version of OppF does not react well with polyclonal antibody against N terminus of OppF. The repeat of this Western Immunoblot in this and our previous publication [24] has shown that truncated version of OppF has only minimal reaction against anti-OppF-N polyclonal antibody. It is postulated that most of epitopes of this antibody are probably conformational (as opposed to linear) and may require of the remaining OppF protein to fully react and provide a readily detectable band on Western Immunoblot.

In bacteria, the Cardiolipin levels have been found to elevate in the stationary growth phase due to up-regulation of Cls activity in response to osmotic stress [25]. The importance of anionic phospholipids cl in the osmotic adaptation and in the membrane structure of *Bacillus subtilis* cultures was demonstrated by impairment of osmotolerance in a Cls mutant (clsA) of this organism. As well as the lack in cl synthesis, this mutant indicated other deficiencies in lipid and fatty acid content compared to the wild-type, signifying a cross-regulation in membrane lipid pathways, critical for the conservation of membrane functionality and integrity [25]. Therefore, it appears that elucidation of the role of Cardiolipin in attenuation of MS-H needs further investigation.

Given that the amino acid substitution in Cls of MS-H^4^ was predicted to change secondary structure of the respective protein compared to that of MS-H and mutation resides in a highly sensitive mutation region, it is likely that this mutation affects the function of the respective protein in MS-H^4^.

The two proteins YbhB and YbcL belong to Raf kinase family and play role in the regulation of protein phosphorylation by kinases in *E. coli* [26]. Phosphorylation and dephosphorylation of proteins play a fundamnetal role in signalling in bacteria [27, 28]. Previous studies have confirmed the significance of the phosphorylation of threonine and histidine residues and serine/threonine kinases which were involved in pathogenicity and stress responses in several prokaryotes [29]. Although the Val74Ile substituion in YbhB/YbcL of isolate 101,546 was unlikey to influence the secondary structure of the respective protein, it was found in a highly sensitive mutation region and therefore could affect the function of this protein.

Earlier studies in our laboratory have revealed that GapA^+^ *M. gallisepticum* (MG) ts-11 vaccine was more immunogenic and induced higher antibody response than the GapA^−^ ts-11 population [30]. In MG, the GapA is determined as the primary cytadhesin molecule and is known to play role in prolonged colonization and survival of MG [31, 32]. Interestingly, isolate 101,546 was recovered from a MS-H vaccinated flock with unusually high systemic antibody response to MS. This isolate had a mutation in NAD-dependent glyceraldehyde-3-phosphate dehydrogenase, located in large pocket regions and found likely to change the secondary structure of the respective protein compared to that of MS-H.

Comparative analysis of the genomes of selected MS isolates from MS-H vaccinated flocks revealed that they were true reisolates of the MS-H vaccine as they had highly similar genome to that of MS-H as opposed to 86,079/7NS. Results of this study also demonstrated that out of 32 mutations found in MS-H genome [12], only four to be reversible (Table 1) after passage in field birds. Thus, the 28 other mutations appear to be stable in MS-H. Of the four unstable mutations, twos (found in the Obg and OppF), were predicted to have some effects on MS virulence.

The mutations which are prone to revert are those that provide advantages to the organism to grow faster or grow in different parts of the respiratory system. For example, reversion mutations in *obg* provide organism higher capacity to live in lower respiratory system or mutation in *oppF* provides organism utilising amino acids more efficiently. These are important to drive reversion to wild-type organism. The mutations that were not found to revert organism to wild-type state probably do not provide the vaccine a significant advantage in vivo.

Given that obtaining pure cultures of the MS-H reisolates characterised here had to undergo multiple steps of growing in liquid and solid media, it may be possible that some of the mutations detected were as a result of in vitro passage. Future studies should therefore target these mutations directly in clinical specimens collected from vaccination chickens.

The data generated in this study also set the foundation for future research aiming to develop strain identification tests that reliably distinguish MS-H from other MS strains that possess identical *vlhA* gene sequence. Furthermore, using a set of mutations found here, it may be possible to correlate results emerging from genotyping techniques to variations in characteristics of MS isolates.

## Conclusion

Results of this study reveal that most of the MS-H mutations are stable after passage in vaccinated chickens. Characterisation of stable mutations observed in MS-H could be applied to develop rapid diagnostic techniques for differentiation of vaccine from field strains or *ts-* MS-H reisolates.

## Methods

### MS strains, growth conditions, and DNA extraction

All MS-H isolates used in this study (Table 3) were made from flocks vaccinated with MS-H at various times after vaccination. All initial swab cultures were cloned by selection of individual colonies three times. The MS-H isolates were grown in mycoplasma broth supplemented with 10% swine serum (Sigma-Australia) and 0.01% (w/v) of nicotinamide adenine dinucleotide (NAD) (Sigma-Australia) [33] at 37 °C in a 50 mL final volume until late logarithmic phase (approximately pH 6.8). Cells were collected followed by extraction of genomic DNA as described previously [12]. The DNA concentration was measured using the optical density at 260 nm (OD_260_) using a NanoDrop™ 2000c spectrophotometer (Thermo Fisher Scientific, Waltham, MA, USA) and purity was evaluated by calculating the OD_260/280_ ratio. The integrity of DNA was assessed using chromatography through 0.8% agarose gel and DNA products were stored at − 80 °C until use.

**Table 3:**
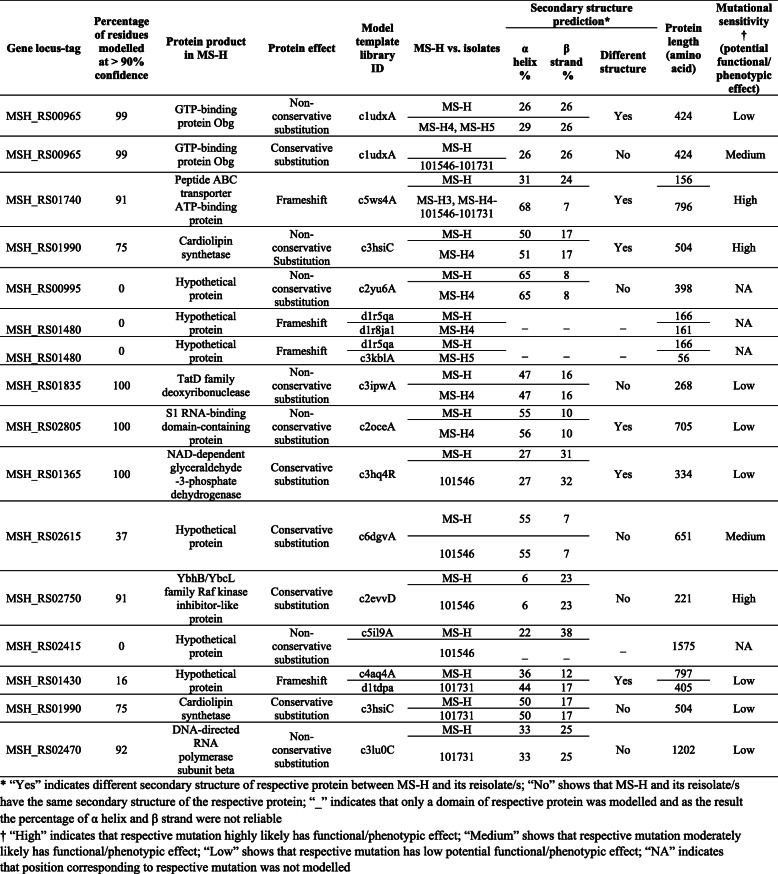
Summary of the isolates examined in this study

### Next-generation sequencing (NGS)

NGS of all MS-H field isolates was performed using Paired-end 125-bp reads by the Illumina MiSeq platform at the Australian Genome Research Facility Ltd. (AGRF, Melbourne, VIC, Australia).

### De novo assembly and sequence analysis

SPAdes assembler version 3.10.0 (Geneious® version 11.1.3) was used to perform De Novo assembly of contiguous sequences. To visualize overall sequence similarity and identify genomic organisation between the MS-H and its field isolates, the contigs were aligned to MS-H genome (GenBank accession number CP021129) as reference using Mauve (Mauve Contig Mover (MCM)), Geneious®. The MCM aligns a draft genome to a reference sequence and orders the contigs in the draft genome according to their position along the reference genome [34, 35].

The resulting contigs and Illumina short reads were mapped to the MS-H genome using Geneious as mapper in Geneious®. Subsequently the alignments were subjected to single nucleotide polymorphism (SNP) and insertion/deletion (indel) analysis. To detect SNPs and indels, ‘Find Variations/SNPs’ in Geneious® was used.

The genome sequence of strain 86,079/7NS (GenBank accession numbers NZ_CP012624) was also included as reference for analysis of SNPs.

### Phylogenetic analysis

To establish the relationship of MS-H isolates (GenBank accession number PRJNA649354), MS-H (GenBank accession number CP021129.1) and 86,079/7NS (GenBank accession number CP012624.1), their whole genome sequence were analysed using maximum likelihood and Neighbor Joining (NJ) methods and the DNA evolutionary models including GTR+ G+ I (GTR: General Time Reversible; G: Gamma distribution; I: evolutionary invariable) and HKY85 (Hasegawa-Kishino-Yano) employing two programs REALPHY (version 1.12) [36] and MEGA (version 10) [37].

### Homology modelling of proteins vary between isolates

The Phyre2 (protein homology/analogy recognition engine V 2.0) web portal for protein modelling, prediction and analysis (http://www.sbg.bio.ic.ac.uk/~phyre2/html/page.cgi?id=index) [38] was used for homology modelling of proteins deduced from genes harboured SNP and indel variants in MS-H field isolates. Intensive mode of modelling was selected which performs complete modelling of the entire protein using multiple templates and ab initio techniques. Furthermore, the resultant modelled protein was subjected to Phyre investigator for more in-depth analysis [39].

The crystal structure of the GTP-binding protein Obg from *Thermus thermophilus* (protein data bank (PDB) ID: c1udxA), ATP-binding/permease from *Acinetobacter baumannii* (PDB ID: c5ws4A), Cardiolipin synthetase (Cls) from (PDB ID: c3hsiC), hydrolase TatD family protein from *Entamoeba histolytica* (PDB ID: c3ipwA), Tex family protein pa5201 from *Pseudomonas aeruginosa* (PDB ID: c2oceA), Glyceraldehyde-3-phosphate2 dehydrogenase from *Staphylococcus aureus* (PDB ID: c3hq4R), Pebp-like protein hp02182 from *Helicobacter pylori* (PDB ID: c2evvD), and DNA-directed RNA polymerase subunit beta from *Escherichia coli* (PDB ID: c3lu0C) were determined and used as homology models for Obg, OppF_,_ Cls, TatD deoxyribonuclease, S1 RNA-binding domain, NAD-dependent glyceraldehyde-3-phosphate dehydrogenase, YbhB/YbcL Raf kinase inhibitor and DNA-directed RNA polymerase subunit beta, respectively.

### Detection of OppF expression in MS strains/isolates

One ml volumes of mycoplasma broth were inoculated with 1/10 dilution of MS strains/isolates (Table 3) and grown to late exponential phase (~ pH 6.8). The cells were treated as described previously [24] and subjected to sodium dodecyl sulfate–polyacrylamide gel electrophoresis (SDS-PAGE) followed by Immunoblotting with mono-specific rabbit sera raised against N terminus of OppF [24].

The MS 94011/V-18d and 86,079/7NS possessing full-length *oppF*, MS-H possessing truncated *oppF*, and recombinant purified OppF [24] were used as controls.

## Data Availability

The datasets generated and/or analysed during the current study are available in the GenBank repository. The Sequence Read Archive (SRA) of the five selected field reisolates of vaccine strain MS-H has been deposited in GenBank under accession number PRJNA649354 [MS-H^3^: SRX8841481; MS-H^4^: SRX8841482; MS-H^5^: SRX8841483; 101564: SRX8841484 and 101731: SRX8841485]. The genome sequences of MS-H and 86079/7NS were retrieved from GenBank (accession numbers CP021129.1 and CP012624.1).

## Abbreviations

MS: *Mycoplasma synoviae*
*ts +*: Temperature sensitive
*ts-*: Non- temperature sensitive
*opp*: Oligopeptide permease
CDS: Coding DNA sequence
w: Wild-type
v: Vaccine-type
MG: *M. gallisepticum*
NGS: Next-generation sequencing
MCM: Mauve Contig Mover
NJ: Neighbor Joining
GTR+ G+ I: General Time Reversible+ Gamma distribution+ evolutionary invariable
HKY85: Hasegawa-Kishino-Yano
Phyre2: Protein homology/analogy recognition engine V 2.0
PDB: Protein data bank
PBS: Phosphate buffered saline
SDS: Sodium dodecyl sulfate
SDS-PAGE: Sodium dodecyl sulfate–polyacrylamide gel electrophoresis
