# Correction to: Preliminary comparative analysis of the genomes of selected field reisolates of the *Mycoplasma synoviae* vaccine strain MS-H reveals both stable and unstable mutations after passage in vivo

**DOI:** 10.1186/s12864-020-07067-y

**Published:** 2020-10-16

**Authors:** Somayeh Kordafshari, Pollob Shil, Marc S. Marenda, Olusola M. Olaogun, Barbara Konsak-Ilievski, Jillian Disint, Amir H. Noormohammadi

**Affiliations:** Asia Pacific Centre for Animal Health, Faculty of Veterinary & Agricultural Sciences, The University of Melbourne, Werribee, Victoria 3030 Australia

**Correction to: BMC Genomics (2020) 21:598**

**https://doi.org/10.1186/s12864-020-06995-z**

Following the publication of the original article [1], it was noted that due to a typesetting error the tables were ordered incorrectly. Tables 1, 2, and 3 were given as Table 2, 3 and 1 respectively.

The original article has been corrected.
